# Opium and Belladonna

**Published:** 1870-06

**Authors:** 


### Selections.

---

Opium and Belladonna.—The antagonism of these drugs is so important to be established, that we take the following cases from the *Trans. Pa. Med. Soc.*

Three cases following are given as contributions toward the subject of the antagonism of opium and belladonna, as well as opium and stramonium. The first and second cases occurred in the practice of Dr. James S. Carpenter, of Pottsville; the third in that of Dr. Krecker, of Cressona. It is believed they will be useful as helping to give data for clinical proof of this antagonism:

Case 1. J. M., mechanic, generally healthy, took for a cold, by mistake, a strong infusion of the leaves of stramonium. His wife was in the habit of giving him a bowl of boneset tea for his colds, and, by mistake, gave the Jamestown weed, nor was the error discovered until he became delirious and insensible.

I was called to see him an hour after he had taken the poison, and found him cold, clammy, almost pulseless, delirious, restless, wanting to get out of bed but unable to stand, with convulsive motions of the lower extremities, inability or great difficulty in swallowing, pupil dilated to the fullest extent, loss of vision, catching at his throat.

I attempted to give him emetics, but did not succeed, applied external stimulants and heat without benefit. Taking into consideration the similarity of symptoms with those of poisoning from belladonna, I resolved to administer the sulphate of morphia hypodermically. Accordingly, I injected about half a drachm of the officinal solution into both arms. In half an hour the convulsions ceased, the delirium subsided, and he slept most of the night, and was able to take some nourishing drinks. The only trouble for some days was dysuria, with some dimness of vision.

Case 2. J. G. B., æt. sixty-five years of age, subject to rheumatic gout, took about forty drops of fluid extract of belladonna for pain in the knee. I was called to see him in two hours after taking the medicine. Found him with a cold, clammy skin, weak and thready pulse, dilated pupils, great difficulty of deglutition, comatose and convulsed. My friend Dr. Palmer had administered emetics and other remedies without effect, and the patient seemed almost in a hopeless condition.

We gave him a teaspoonful of tr. opii every hour, until he had taken three doses, when the quantity was diminished one-half, and the intervals lengthened. After the second dose of laudanum had been given, the convulsions ceased, the delirium lessened, and he sank gradually into a quiet sleep, the pupils contracted, the skin became warm, and all alarming symptoms subsided. The delirium continued in a mild form for twelve or fifteen hours longer; but after sleeping, with slight intermissions, all night, he awoke the next morning in his right mind.

I think there can be no doubt that his life was saved by the administration of the opium, of which he took altogether about half an ounce. Emetics, external stimulants and other appropriate treatment has been tried without benefit, before giving the opium, and the effect of the latter was so marked in alleviating the urgent symptoms, that I am satisfied that in this case the opium acted as a direct antidote to the poison of the belladonna.

Case 3. I was called, April 2d, 1869, to see E. B., a robust male infant of three months, under the influence of an over-dose of laudanum, administered by the mother to quiet a little restlessness.

No reliable information could be obtained in reference to the quantity. The little patient was found in deep coma, the surface pale, lips and angles at alæ nasi livid, muscles relaxed, breathing slow, labored and stertorious. The iris was strongly contracted, a veritable pin-hole pupil; the heart's action was feeble.

Gave immediately six drops of tr. belladonnæ, time, 2:45 P. M. Cold water was dashed on the surface, and sinapisms applied to the extremities, while respiration, which was at times quite feeble, was occasionally assisted artificially.

In forty minutes the iris had become a little more relaxed, and a better color appeared on the surface. Gave five drops tr. belladonnæ. Patient continued to improve, the circulation getting better, muscular action returning, and the pupils becoming more dilated.

At 5 P. M. Dr. J. G. Kœhler arrived, and advised three drops more of tr. belladonnæ, with applications of ice to the spine. At 7 P. M. gave three drops tr. belladonnæ, and at 7:45 left the patient with the pupils of full size, the circulation and respiration good, readily roused, crying lustily.

Called next day and found the little patient bright and lively.

